# Supplementary material for: Wheat powdery mildew resistance gene Pm13 encodes a mixed lineage kinase domain-like protein
Source: Nat Commun. 2024 Mar 19;15:2449. doi: 10.1038/s41467-024-46814-7 (PMC10951266; doi:10.1038/s41467-024-46814-7)
Supplement: Supplementary file 1 — Supplementary Information [file 41467_2024_46814_MOESM1_ESM.pdf]

Wheat powdery mildew resistance gene *Pm13* encodes a mixed lineage kinase domain-like protein

Li *et al.*

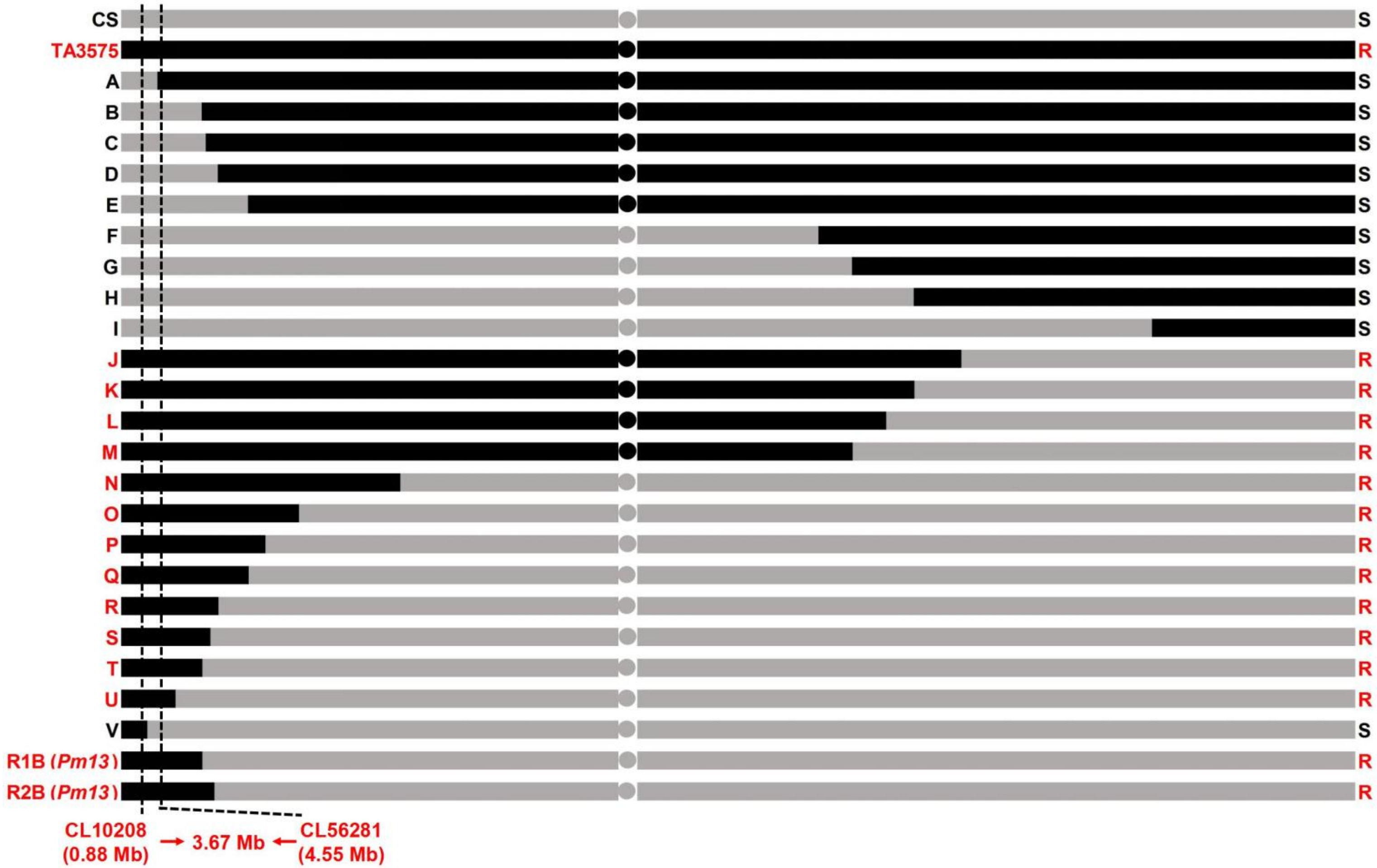

Supplementary Fig. 1. Initial mapping of *Pm13a*. A-V: Twenty-two different types of CS-*Ae. longissima* 3S^l#2 recombinants. R1B: CS-*Ae. longissima* T3S^l#1S-3BS.3BL recombinant. R2B: CS-*Ae. longissima* T3S^l#1S-3DS.3DL recombinant. R indicated resistance to powdery mildew, S indicated susceptible to powdery mildew.

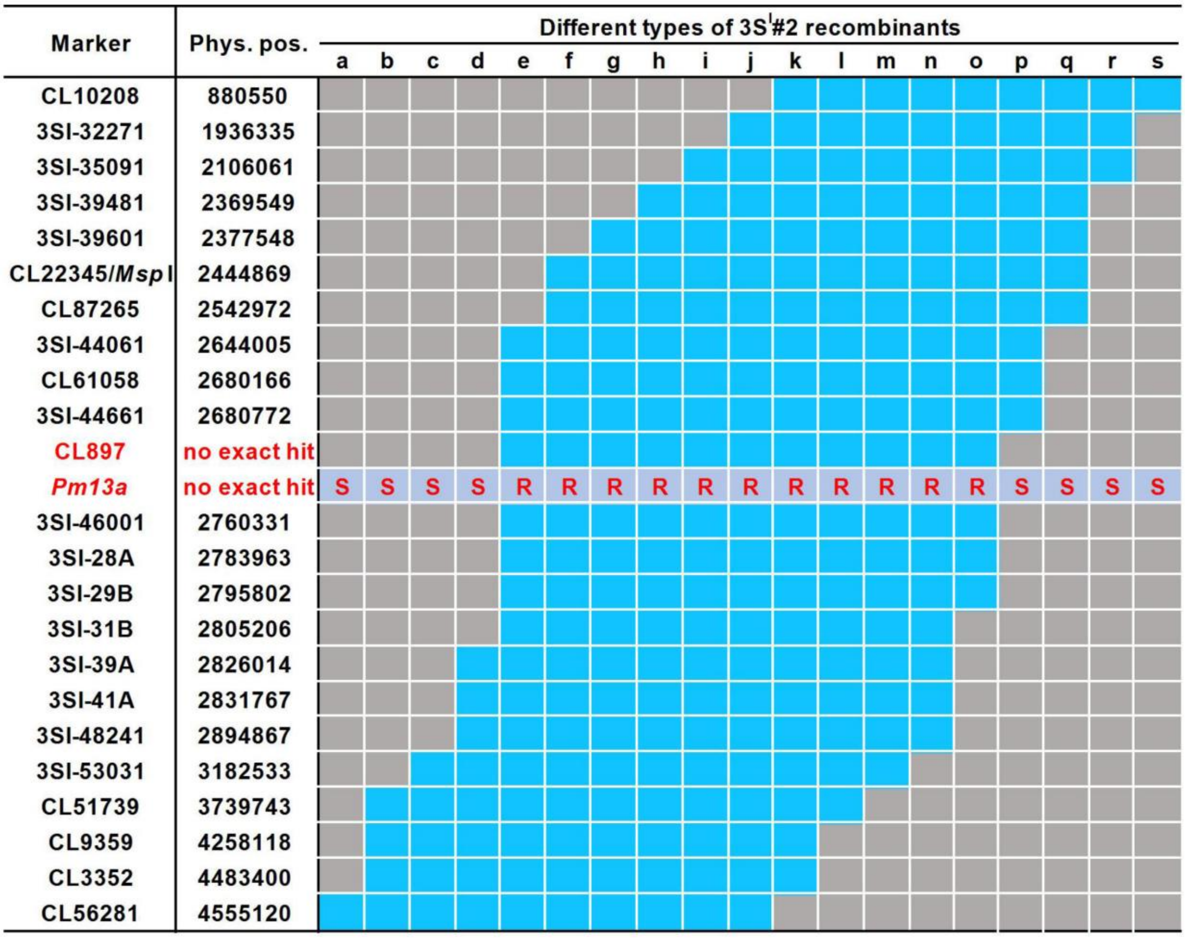

Supplementary Fig. 2. High-resolution mapping of *Pm13a*. a-s: Nineteen different types of CS-*Ae. longissima* 3S^{l}#2 recombinants. Phys. Pos.: The physical position of 3S^{l}#2-specific molecular marker on *Ae. longissima* TL05 reference genome. The F_2 progenies of 19 different types of wheat-*Ae. longissima* 3S^{l}#2 recombinants were inoculated with *Bgt* isolate E09 for powdery mildew resistance evaluation. R: resistance to powdery mildew, S: susceptible to powdery mildew. Blue box indicated the presence of *Ae. longissima* 3S^{l}#2-specific molecular markers, while gray box indicated the absence of *Ae. longissima* 3S^{l}#2-specific molecular marker.

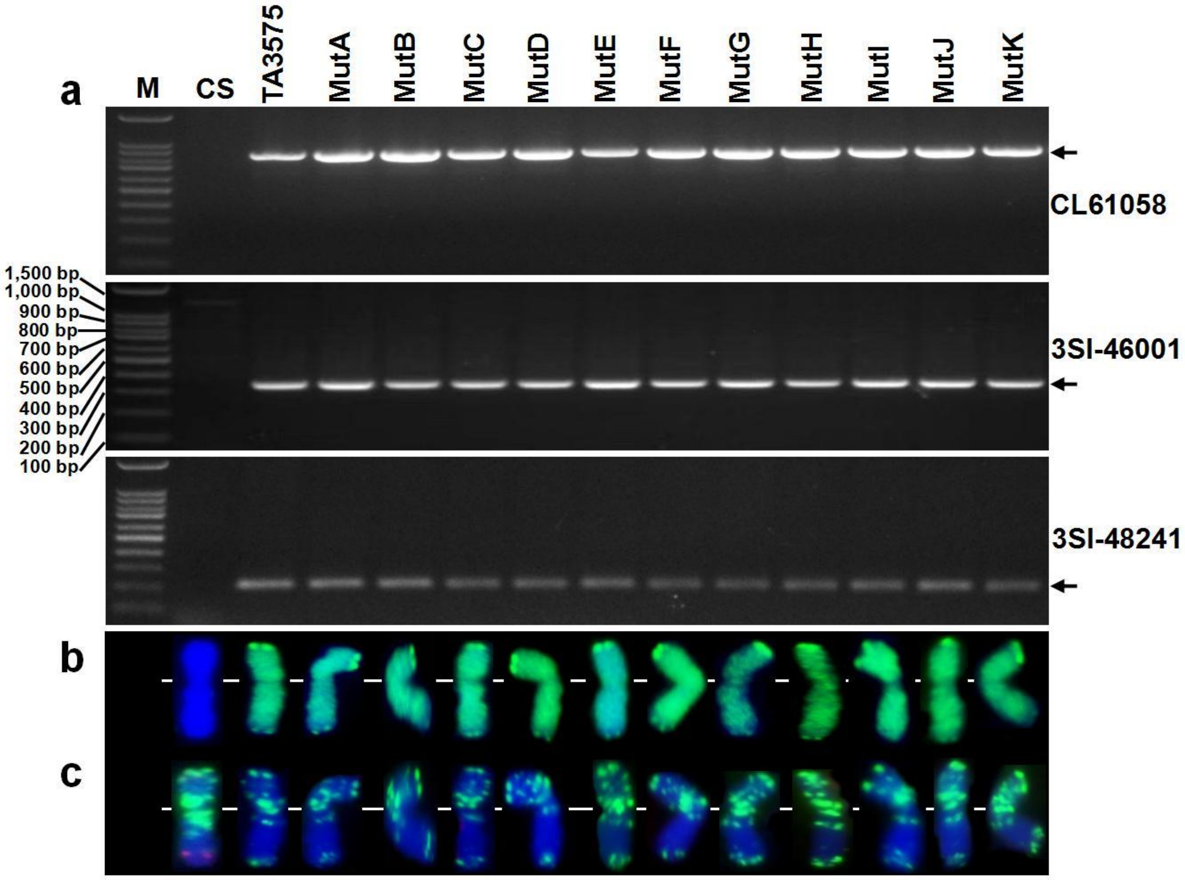

Supplementary Fig. 3. 3S^l#2-specific molecular markers, GISH and FISH validation of 11 TA3575 EMS susceptible mutants. a Molecular markers analysis of 11 TA3575 EMS susceptible mutants. M, 100 bp Ladder DNA marker. Arrows pointed to the polymorphic bands of the 3S^l#2-specific molecular markers present in TA3575 and 11 mutants and absent in CS. **b** GISH patterns of 11 TA3575 EMS susceptible mutants. Total gDNA of *Ae. longissima* was labeled with fluorescein-12-dUTP and visualized with green fluorescence. Wheat chromatin was counterstained with 4',6-diamidino-2-phenylindole (DAPI) and visualized with blue fluorescence. **c** FISH patterns of 11 TA3575 EMS susceptible mutants. FAM-modified oligonucleotides (pSc119.2-1 and $(GAA)_{10}$) were in green color. TAMRA-modified oligonucleotides (pAs1-1, pAs1-3, pAs1-4, pAs1-6, AFA-3 and AFA-4) were in red color. Wheat chromatin was counterstained with DAPI and visualized with blue fluorescence.

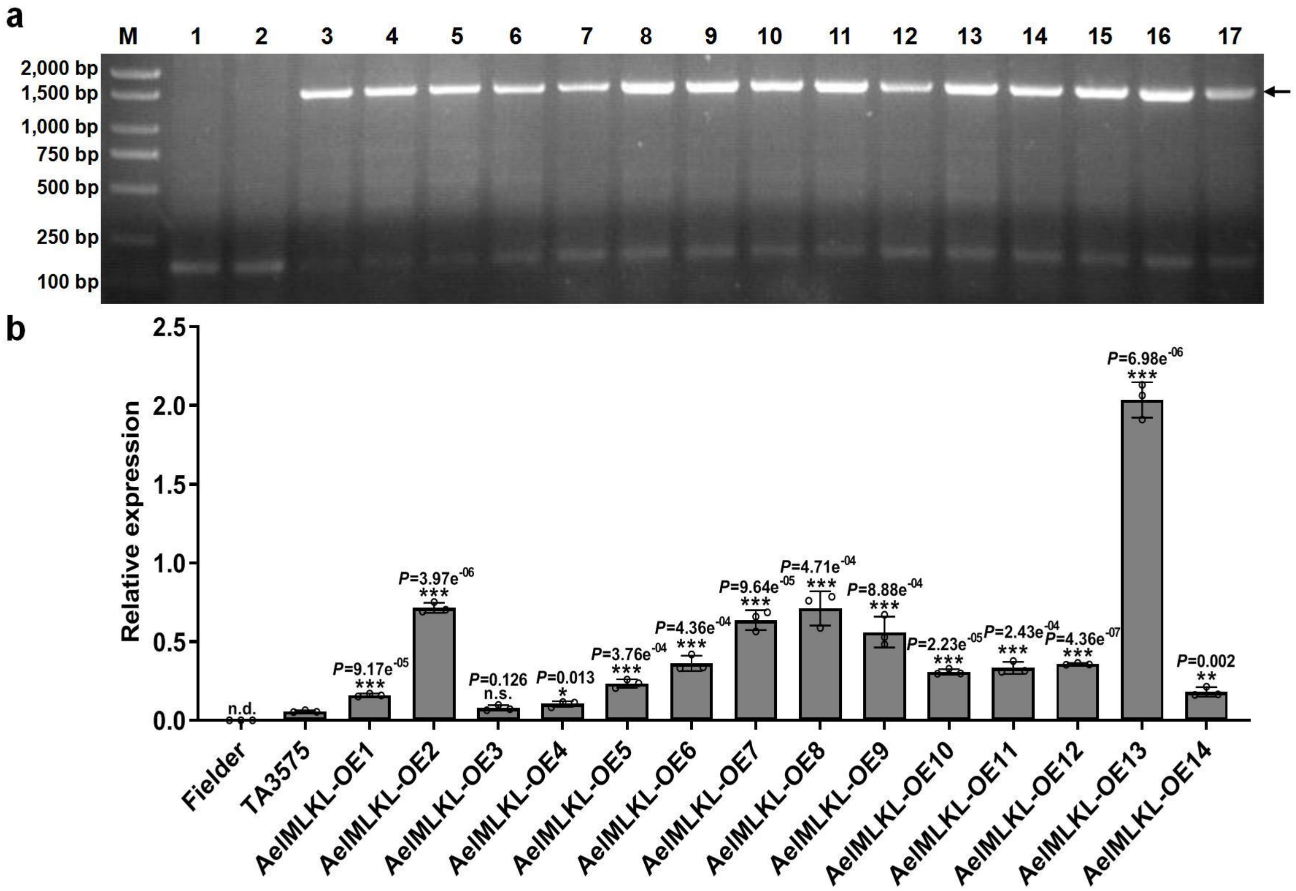

Supplementary Fig. 4. Molecular detection of T_0 and T_1 transgenic plants with *AelMLKL* gene mediated by *Agrobacterium tumefaciens*. a Detection of putative T_0 transgenic plants by PCR amplification of *AelMLKL* gene using primer AelMLKL_OE. M, DL2000 Plus DNA marker; 1, common wheat CS; 2, common wheat cultivar Fielder; 3, positive control of expression vector pWMB110 containing the full sequence of *AelMLKL*; 4-17, 14 individual T_0 transgenic plants. Arrows pointed to the polymorphic bands of molecular marker AelMLKL_OE. **b** The transcriptional levels of *AelMLKL* gene in T_1 transgenic positive plants derived from selfing of different T_0 individuals were examined before inoculation at the seedling stage. The T_1 transgenic positive plants were selected by PCR using specific marker AelMLKL_OE. The expression analysis is based on three biological replicates with five leaves from five positive individuals per sample. Fielder was used as negative control. TA3575 was used as positive control. The values are the mean ± SD (two-sided *t*-test, n = 3 biologically independent experiments, $^{***}P < 0.001$, $^{**}P < 0.01$, $^{*}P < 0.05$, N.D., not detected, N.S., no significant). Source data are provided as a Source Data file.

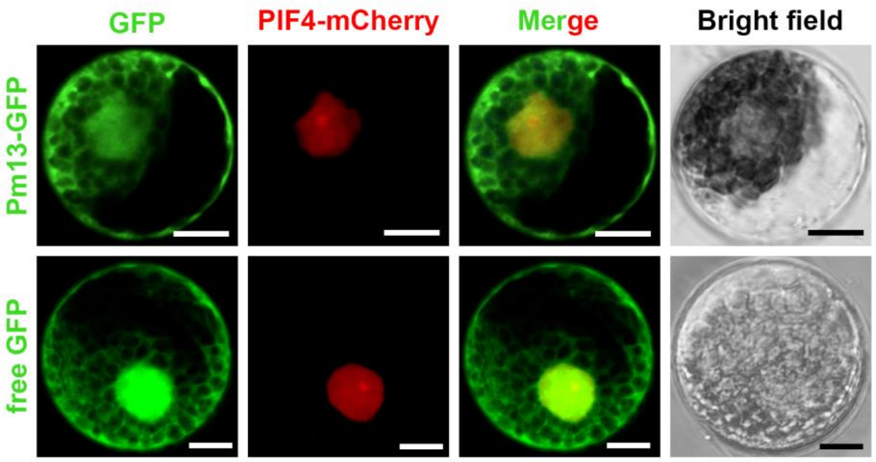

Supplementary Fig. 5. Subcellular localization of the Pm13 protein. The *Pm13-GFP* construct was co-transformed with nucleus marker plasmid *AtPIF4-mCherry* into wheat protoplast cells. Bar = 10 μm.

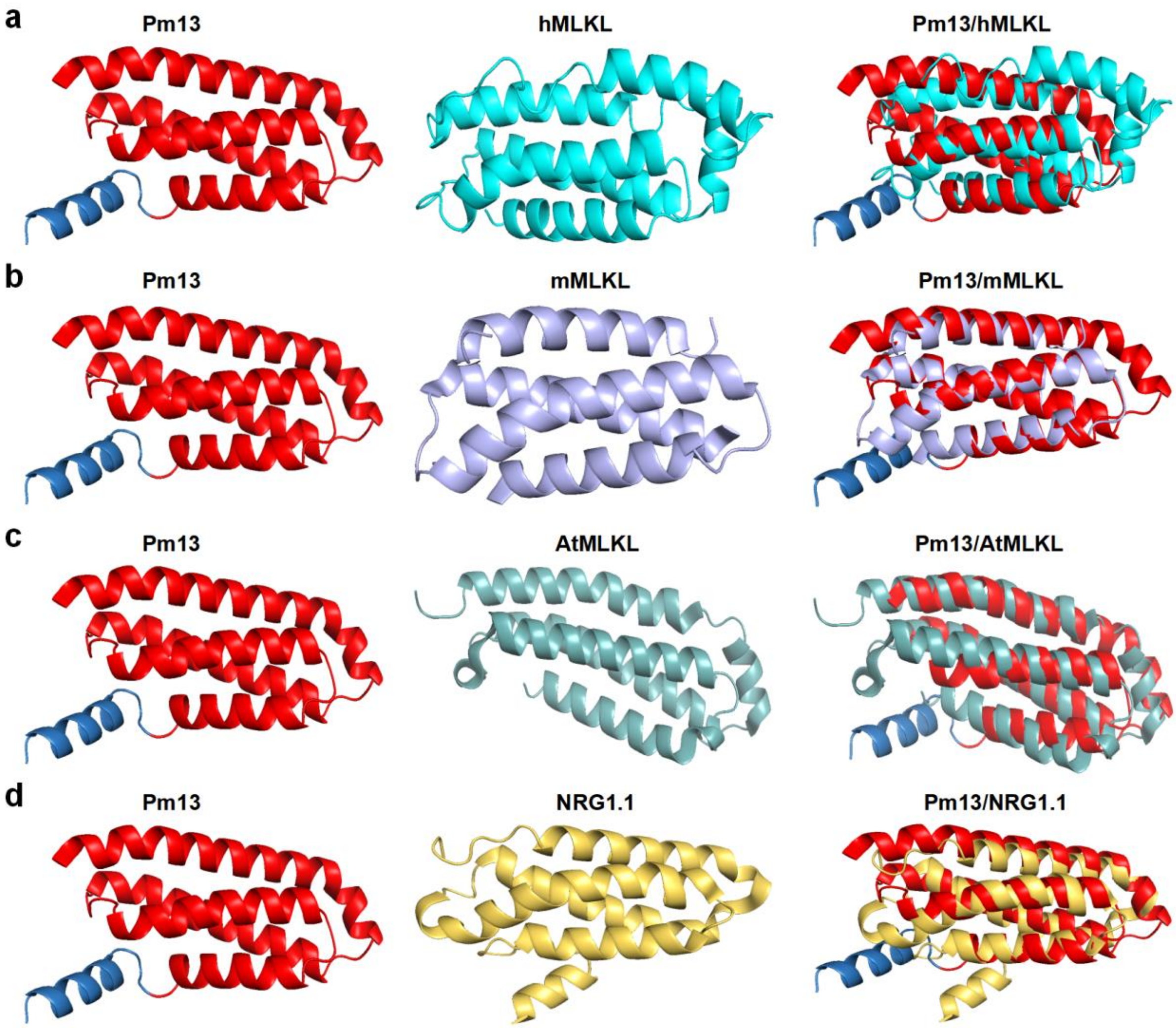

Supplementary Fig. 6. Pm13 MLKL_NTD domain superimposed well with MLKL_NTD domain of human MLKL (hMLKL) and mouse MLKL (mMLKL), DUF1221 domain of *Arabidopsis thaliana* MLKL (AtMLKL), and CC_R domain of *Nicotiana benthamiana* NRG1.1. a Superimposition of the Pm13 MLKL_NTD domain (red) with hMLKL MLKL_NTD domain (cyan, PDB ID code 2MSV). **b** Structural overlay of the Pm13 MLKL_NTD domain (red) with mMLKL MLKL_NTD domain (lightblue, PDB ID code 4BTF). **c** Superposition of the Pm13 MLKL_NTD domain (red) and AtMLKL DUF1221 domain (lightteal, PDB ID code 6KA4). **d** Superposition of the Pm13 MLKL_NTD domain (red) and NRG1.1 CC_R domain (paleyellow, PDB ID code 7L7V).

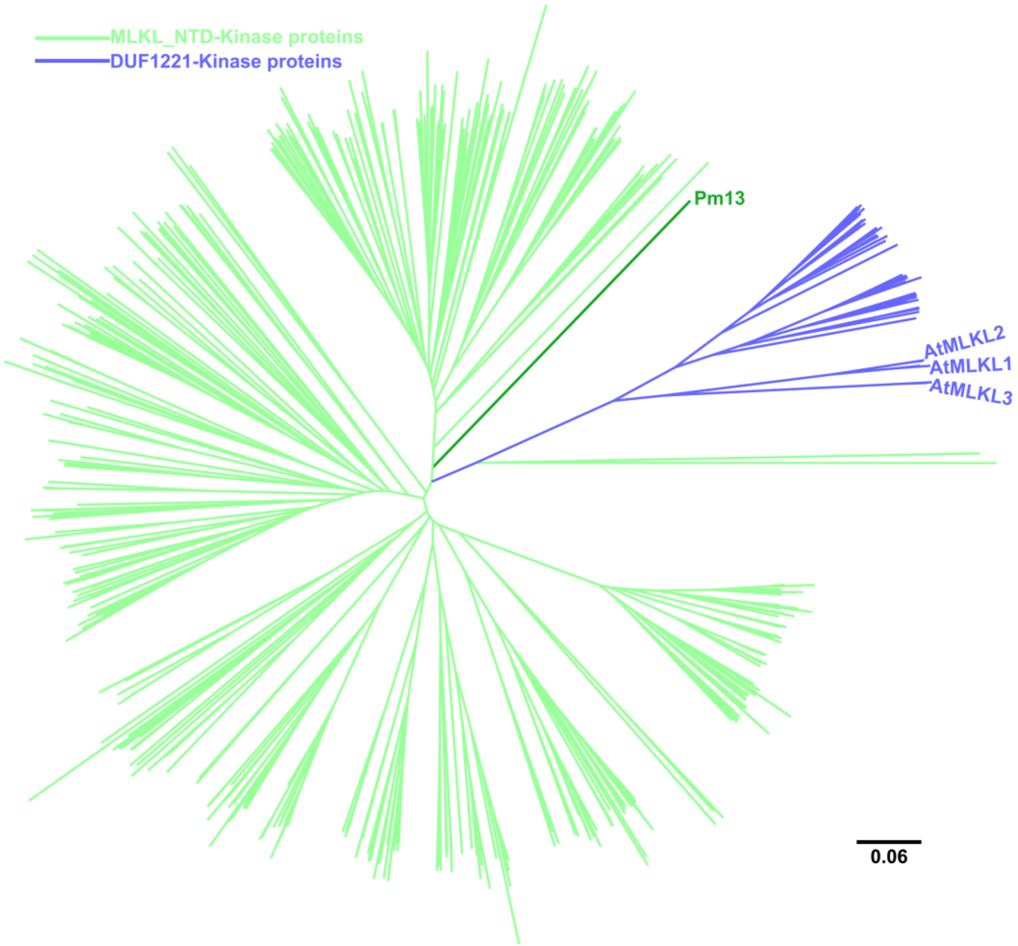

Supplementary Fig. 7. Phylogenetic analysis of 714 MLKL_NTD-Kinase proteins and 97 DUF1221-Kinase proteins from *Poaceae* and *Arabidopsis* in Interpro database (https://www.ebi.ac.uk/interpro), three MLKL proteins (AtMLKL1, AtMLKL2 and AtMLKL3) reported by Mahdi *et al.*[1], and Pm13. The phylogenetic tree was constructed using MEGA7 software with the neighbour-joining method and drawn with iTOL (https://itol.embl.de/).

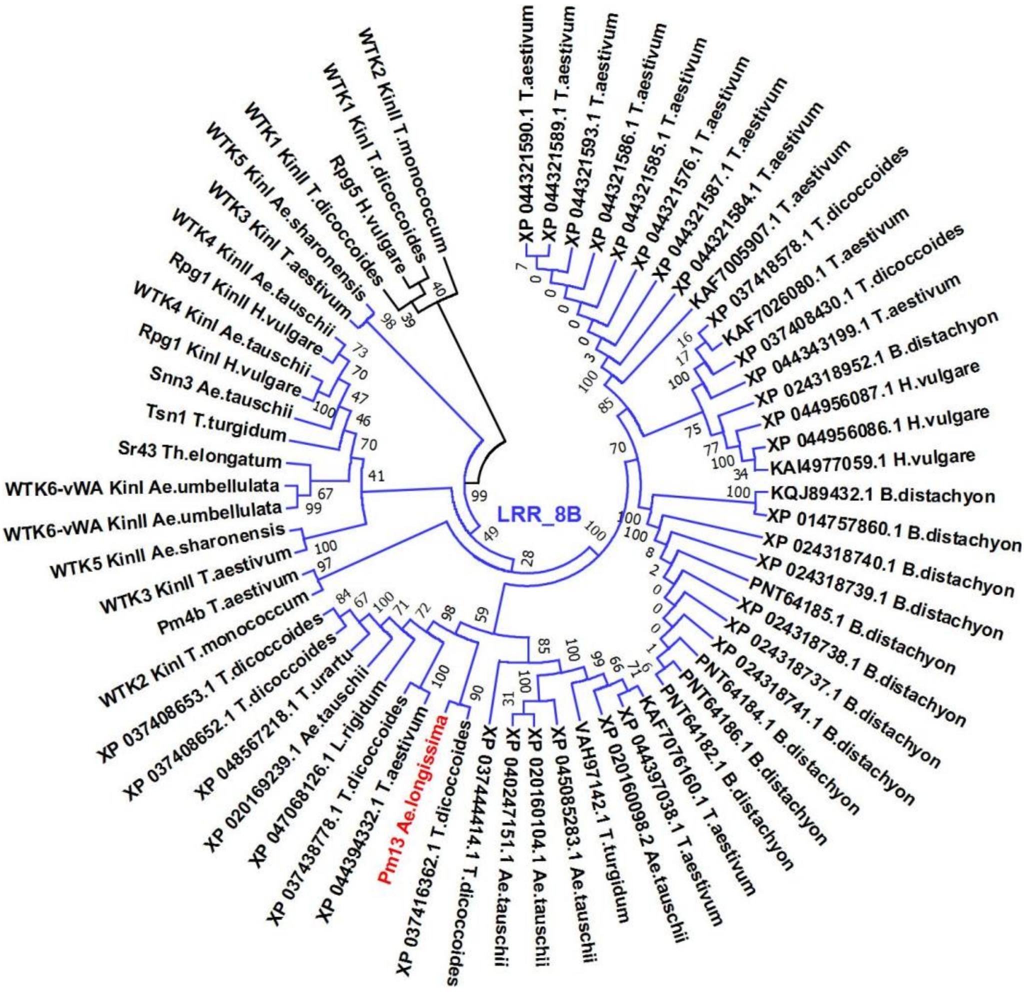

Supplementary Fig. 8. Phylogenetic tree of the kinase domain of Pm13, its homologs with best hits from NCBI protein database (coverage > 94% and identity > 60%), and recently cloned KFPs. The phylogenetic tree was constructed using MEGA7 software with the neighbour-joining method and drawn with iTOL (https://itol.embl.de/).

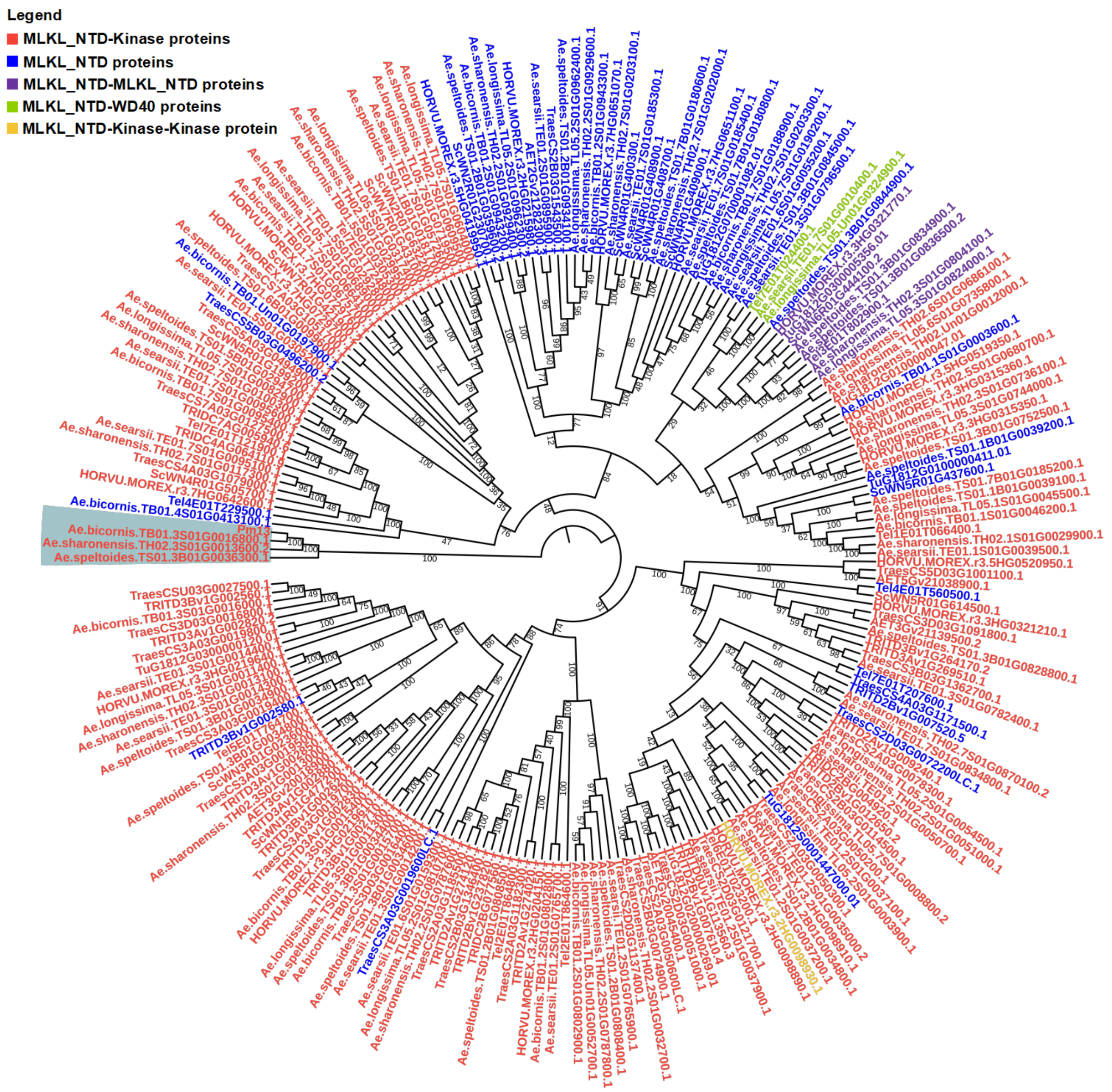

Supplementary Fig. 9. Phylogenetic analysis of 220 MLKL_NTD domain containing proteins from Triticeae tribe at WheatOmics (http://wheatomics.sdau.edu.cn/). The phylogenetic tree was constructed using MEGA7 software with the neighbour-joining method and drawn with iTOL (https://itol.embl.de/).

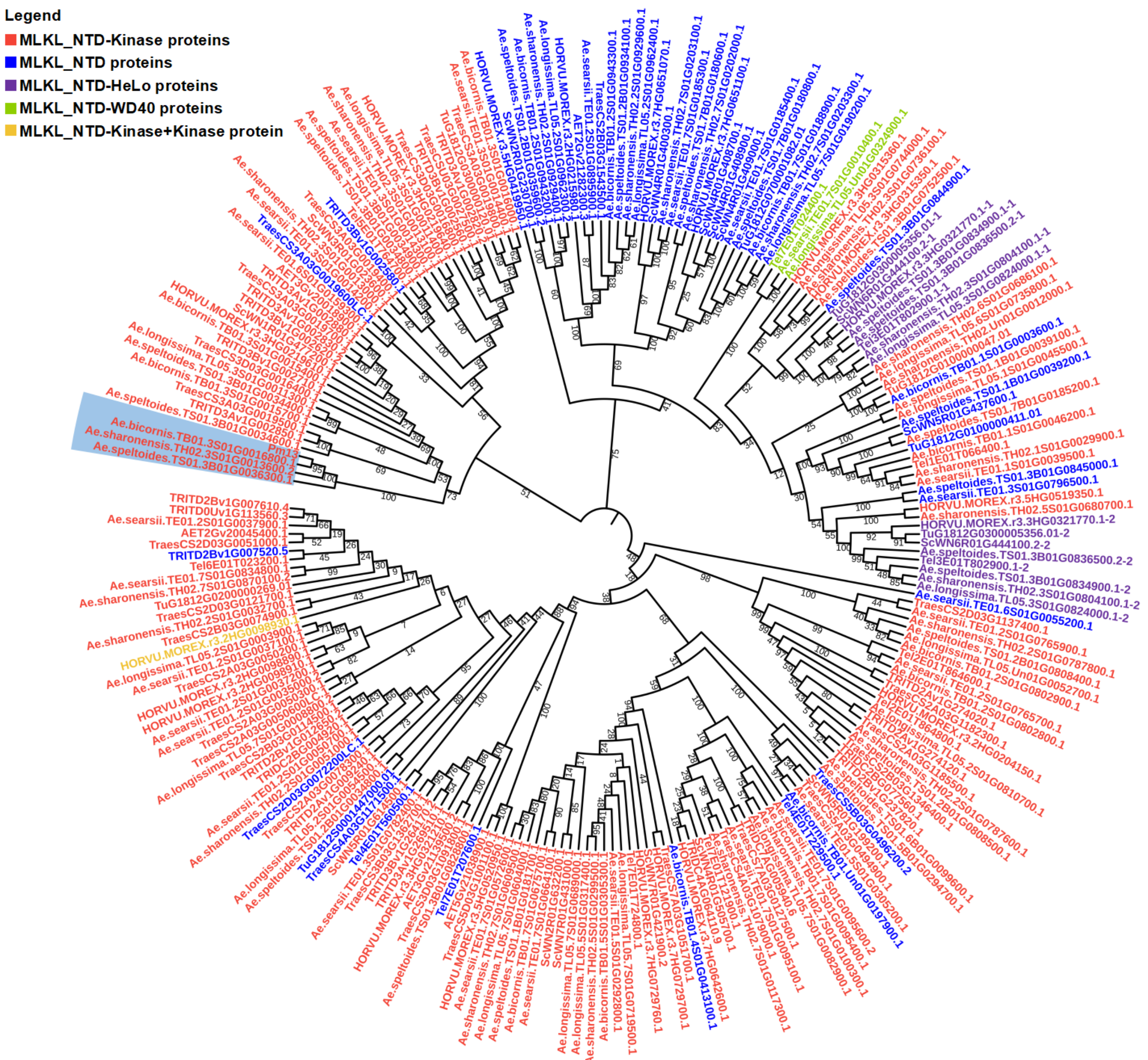

Supplementary Fig. 10. Phylogenetic tree analysis with 228 MLKL_NTD domains from 220 MLKL_NTD domain containing proteins in Triticeae tribe at WheatOmics (http://wheatomics.sdau.edu.cn/). The phylogenetic tree was constructed using MEGA7 software with the neighbour-joining method and drawn with iTOL (https://itol.embl.de/).

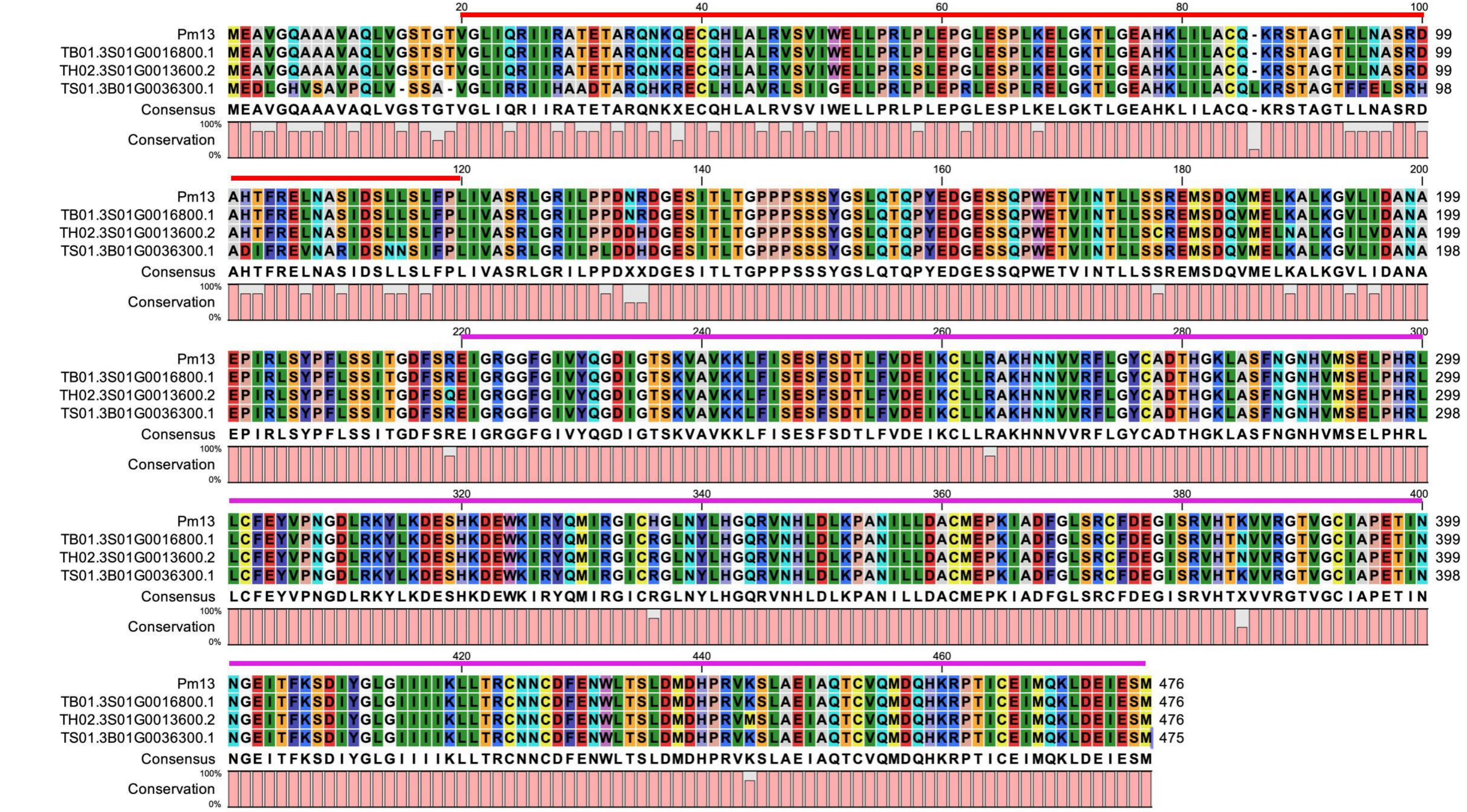

Supplementary Fig. 11. Protein sequence alignment of Pm13 and Pm13 orthologs. Predicted MLKL_NTD (red) and STK (magenta) domains are indicated above the sequence.

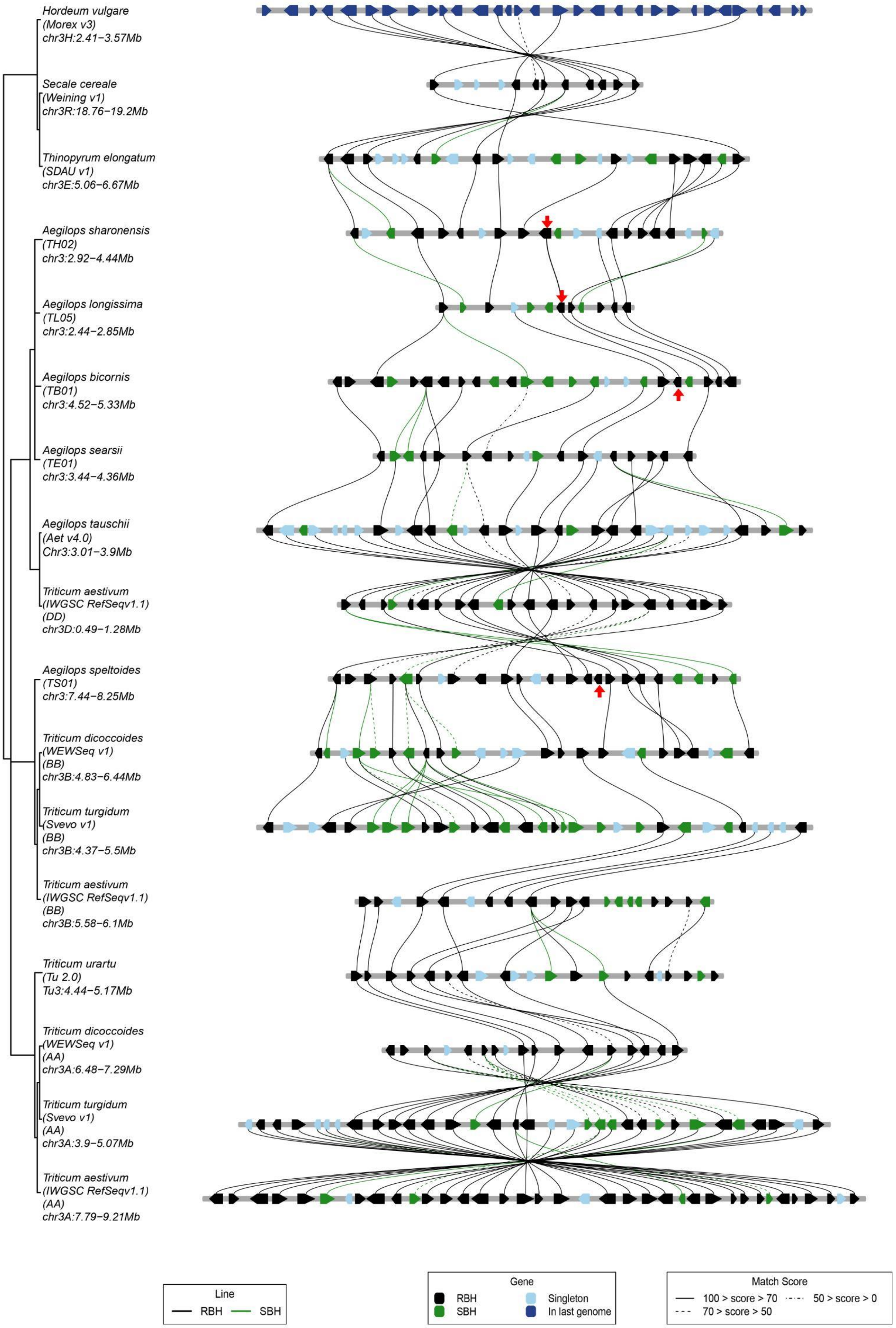
Hordeum vulgare (Morex v3) chr3H:2.41−3.57Mb
Secale cereale (Weining v1) chr3R:18.76−19.2Mb
Thinopyrum elongatum (SDAU v1) chr3E:5.06−6.67Mb
Aegilops sharonensis (TH02) chr3:2.92−4.44Mb
Aegilops longissima (TL05) chr3:2.44−2.85Mb
Aegilops bicornis (TB01) chr3:4.52−5.33Mb
Aegilops searsii (TE01) chr3:3.44−4.36Mb
Aegilops tauschii (Aet v4.0) Chr3:3.01−3.9Mb
Triticum aestivum (IWGSC RefSeqv1.1) (DD) chr3D:0.49−1.28Mb
Aegilops speltoides (TS01) chr3:7.44−8.25Mb
Triticum dicoccoides (WEWSeq v1) (BB) chr3B:4.83−6.44Mb
Triticum turgidum (Svevo v1) (BB) chr3B:4.37−5.5Mb
Triticum aestivum (IWGSC RefSeqv1.1) (BB) chr3B:5.58−6.1Mb
Triticum urartu (Tu 2.0) Tu3:4.44−5.17Mb
Triticum dicoccoides (WEWSeq v1) (AA) chr3A:6.48−7.29Mb
Triticum turgidum (Svevo v1) (AA) chr3A:3.9−5.07Mb
Triticum aestivum (IWGSC RefSeqv1.1) (AA) chr3A:7.79−9.21Mb
Line
RBH
SBH
Gene
RBH
SBH
Singleton
In last genome
Match Score
100 > score > 70
70 > score > 50
50 > score > 0

Supplementary Fig. 12. Collinearity of the *Pm13* genomic regions in Triticeae tribe. Collinearity analysis was performed using the online tool Triticeae-GeneTribe[98]. Orthologous genes are linked by lines, and *Pm13* orthologous genes are shown in red arrows. The *Pm13* orthologs were present only in *Aegilops bicornis* (TB01) (S^bS^b), *Ae. sharonensis* (TH02) ($S^{sh}S^{sh}$) and *Ae*. *speltoides* (TS01) (SS). RBH (reciprocal best hit), SBH (single-side best hit) and singleton indicated three different types of homologous relationships. RBH: gene pairs belonging to the Reciprocal Best Hits; SBH: gene pairs belonging to the Single-side Best Hits, where RBH is not found but the best matching gene is found; Singleton: the genes with no homologous genes. In last genome: The last genome in the picture. Because the Triticeae-GeneTribe database uses RBH and SBH data structures, where SBH is unidirectional, the last genome cannot be compared with other genomes when drawing from the bottom up. The left tree is obtained from a pre-computed relationship derived from Triticeae-GeneTribe.

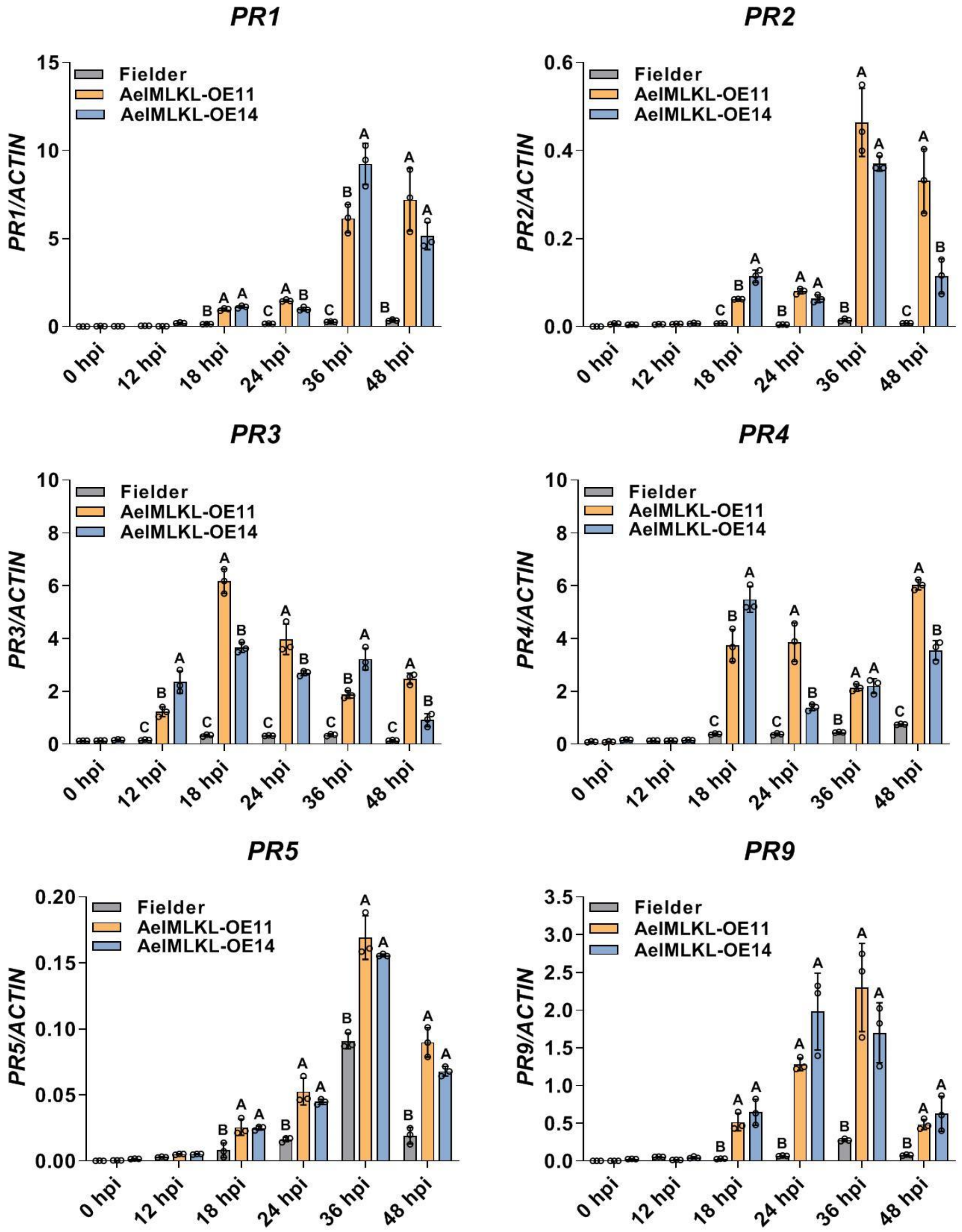

Supplementary Fig. 13. Relative expression level of PR genes from one-week-old *Pm13* transgenic lines AeIMLKL-OE11 and AeIMLKL-OE14 and susceptible control Fielder seeding plants at 0, 12, 18, 24, 36 and 48 hpi with *Bgt* isolate E09. Expression of the *TaActin* gene was used for normalization. The values of qRT-PCR are the mean ± SD (n = 3 biologically independent experiments). Different capital letters above the bar denote highly significant differences (*P* < 0.01) of the mean values by one-way ANOVA and multiple comparisons using Fisher's LSR test. Source data are provided as a Source Data file.

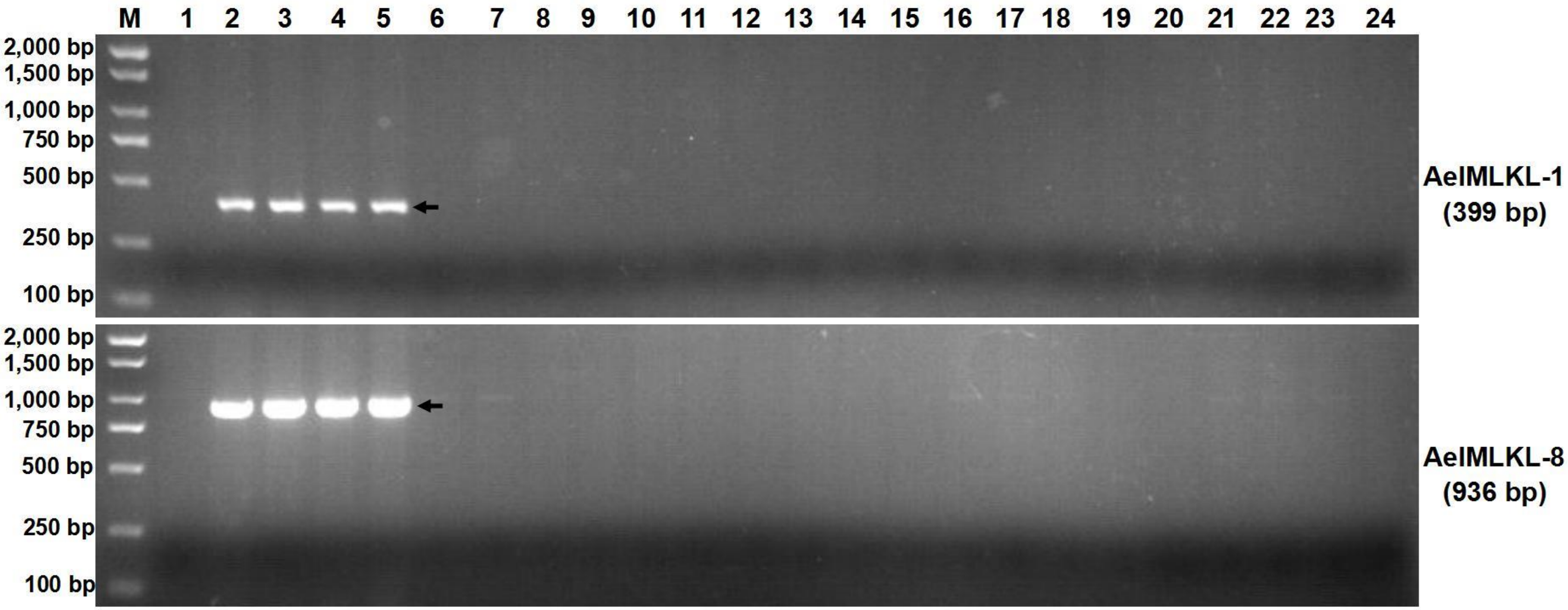

Supplementary Fig. 14. Validation of the usefulness of functional molecular markers AelMLKL-1 and AelMLKL-8 of *Pm13* in partial wheat lines. M, DL2000 Plus DNA marker; 1, CS; 2, CS-*Ae. longissima* $3S^l$#2(3B) disomic substitution line TA3575; 3, CS-*Ae. longissima* $T3S^l$#1S-3BS.3BL recombinant R1B; 4, CS-*Ae. longissima* $T3S^l$#1S-3DS.3DL recombinant R2B; 5, the developed CS-*Ae. longissima* $T3S^l$#2S-3BS.3BL recombinant W12-3 with tiny $3S^l$#2 segment harboring *Pm13*; 6, Bainong 64; 7, Bainong 207; 8, Zhoumai 16; 9, Zhoumai 18; 10, Zhoumai 22; 11, Zhoumai 28; 12, Ping'an 602; 13, Ping'an 0518; 14, Ping'an 901; 15, Yanzhan 4110; 16, Aikang 58; 17, Zhengzhou 366; 18, Xinong 979; 19, Nannong 9918; 20, Tianmin 198; 21, Tianmin 369; 22, Yangmai 5; 23, Yunong 202; 24, Yumai 49-198. Arrows pointed to the polymorphic bands of *Pm13* functional molecular markers AelMLKL-1 and AelMLKL-8.

Supplementary Table 1. Molecular characterization of the *Pm13a* EMS mutant.

Mutant line	Mutation type	Position (cDNA)	Exon	Codon change	Amino acid change	Affected domain	Infection type	Evaluation of powdery mildew
MutA	Missense	743	4	AGT→AAT	S 248 N	STK	4	S
		1112	5	TCG→TTG	S 371 L	STK		
MutB	Missense	1183	6	CCG→TCG	P 395 S	STK	4	S
MutC	Missense	1087	5	CCA→TCA	P 363 S	STK	4	S
MutD	Missense	928	4	CTT→TTT	L 310 F	STK	4	S
MutE	Missense	1172	6	GGA→GAA	G 391 E	STK	4	S
MutF	Missense	272	1	GGC→GAC	G 91 D	MLKL_NTD	4	S
MutG	Missense	995	5	GGA→GAA	G 332 E	STK	4	S
MutH	Missense	556	3	GAA→AAA	E 186 K	Brace	4	S
MutI	Frameshift	277-278 deletion	1	-	-	MLKL_NTD	4	S
MutJ	Missense	1184	6	CCG→CTG	P 395 L	STK	4	S
MutK	Nonsense	1293	7	TGG→TGA	W 431 * stop codon	STK	4	S

Note: S indicated susceptible to powdery mildew. 4 as highly susceptible.

Supplementary Table 2. Powdery mildew reactions of the T_1 transgenic plants to *Bgt* isolate E09.

T_1 transgenic family	Transgenic positive plants			Transgenic negative plants	
	No. of positive plants	No. of resistant plants	No. of susceptible plants	No. of negative plants	No. of susceptible plants
AelMLKL-OE1	14	14	0	2	2
AelMLKL-OE2	13	13	0	3	3
AelMLKL-OE3	13	13	0	3	3
AelMLKL-OE4	8	8	0	8	8
AelMLKL-OE5	14	14	0	2	2
AelMLKL-OE6	10	10	0	6	6
AelMLKL-OE7	11	11	0	5	5
AelMLKL-OE8	12	12	0	4	4
AelMLKL-OE9	13	13	0	3	3
AelMLKL-OE10	14	14	0	2	2
AelMLKL-OE11	11	11	0	5	5
AelMLKL-OE12	11	11	0	5	5
AelMLKL-OE13	9	9	0	7	7
AelMLKL-OE14	11	11	0	5	5

Supplementary Table 3. Allelism test between *Pm13*, *Pm13a*, and *Pm13b*.

**Female**	**Male**	**Total plants of F_2 population**	**Response to powdery mildew inoculation with *Bgt* isolate E09**
R1B (*Pm13*)	TA3575 (*Pm13a*)	2,183	R
TA3575 (*Pm13a*)	R1B (*Pm13*)	2,785	R
TA7545 (*Pm13b*)	R1B (*Pm13*)	2,082	R
R1B (*Pm13*)	TA7545 (*Pm13b*)	2,715	R
TA7545 (*Pm13b*)	TA3575 (*Pm13a*)	2,168	R
TA3575 (*Pm13a*)	TA7545 (*Pm13b*)	2,176	R

Note: R indicated resistance to powdery mildew.

Supplementary Table 4. List of *Pm13* orthologous genes found in different species within the Triticeae tribe.

Species	Gene	Chromosome	Chromosome length	blast_hit_1	blast_hit_2
Aegilops bicornis (cv. TB01)	TB01.3S01G0016800.1	$3S^b$	838613969	5256873	5249683
Aegilops sharonensis (cv. TH02)	TH02.3S01G0013600.2	$3S^{sh}$	800427778	4144675	4137412
Aegilops speltoides (cv. TS01)	TS01.3B01G0036300.1	3S	578812325	8050402	8042742

Supplementary reference

[1] Mahdi, L. K. *et al*. Discovery of a family of mixed lineage kinase domain-like proteins in plants and their role in innate immune signaling. *Cell Host Microbe* **28**, 813–824 (2020).
