# Supplementary material for: Wheat powdery mildew resistance gene Pm13 encodes a mixed lineage kinase domain-like protein
Source: Nat Commun. 2024 Mar 19;15:2449. doi: 10.1038/s41467-024-46814-7 (PMC10951266; doi:10.1038/s41467-024-46814-7)
Supplement: Supplementary file 3 — Description of Additional Supplementary Files [file 41467_2024_46814_MOESM3_ESM.pdf]

Description of Additional Supplementary Files

File Name: Supplementary Data 1

Description: List of PCR primers used for initial mapping of *Pm13a*.

File Name: Supplementary Data 2

Description: Characterization of CS-*Ae. longissima* $3S^l$#2 recombinants using 43 $3S^l$#2-specific molecular markers for initial mapping of *Pm13a*.

File Name: Supplementary Data 3

Description: List of PCR primers used for fine mapping of *Pm13a*.

File Name: Supplementary Data 4

Description: Characterization of CS-*Aegilops longissima* $3S^l$#2 recombinants for fine mapping of *Pm13a*.

File Name: Supplementary Data 5

Description: The information of unigene CL897Contig1 from RNA-Seq of *Pm13a* donor parent TA3575.

File Name: Supplementary Data 6

Description: List of PCR primers used for CDS amplification, marker-assisted selection (MAS), virus-induced gene silencing (VIGS), vectors construction for transgenic and subcellular localization, validation of the presence of transgene, qRT-PCR for *AelMLKL* and *PR* genes.

File Name: Supplementary Data 7

Description: List of *Bgt* isolates used to characterize the resistance spectra of *Pm13*, *Pm13a* and *Pm13b*.

File Name: Supplementary Data 8

Description: Kinase domain comparison analysis of Pm13.

File Name: Supplementary Data 9

Description: The amino acid sequences of 714 MLKL_NTD-Kinase proteins from *Poaceae* and

Arabidopsis in Interpro database.

File Name: Supplementary Data 10

Description: The amino acid sequences of 97 DUF1221-Kinase proteins from *Poaceae* and *Arabidopsis* in InterPro database.

File Name: Supplementary Data 11

Description: Presence of Pm13 kinase homologs in various plant species.

File Name: Supplementary Data 12

Description: The amino acid sequences of 220 MLKL_NTD domain containing proteins across the Triticeae tribe at WheatOmics.

File Name: Supplementary Data 13

Description: MLKL_NTD domain containing proteins in the Triticeae tribe at WheatOmics.

File Name: Supplementary Data 14

Description: The amino acid sequences of 228 MLKL_NTD domains based on 220 MLKL_NTD domain-containing proteins across the Triticeae tribe at WheatOmics.

File Name: Supplementary Data 15

Description: Wheat accessions used for identification of *Pm13*.
